# Supplementary material for: Spontaneous Attenuation of Alcoholic Fermentation via the Dysfunction of Cyc8p in Saccharomyces cerevisiae
Source: Int J Mol Sci. 2023 Dec 25;25(1):304. doi: 10.3390/ijms25010304 (PMC10778621; doi:10.3390/ijms25010304)
Supplement: Supplementary file 1 [file ijms-25-00304-s001.zip › ijms-2780459-supplementary.pdf]

Supplementary Figure S1 (Watanabe *et al.*)

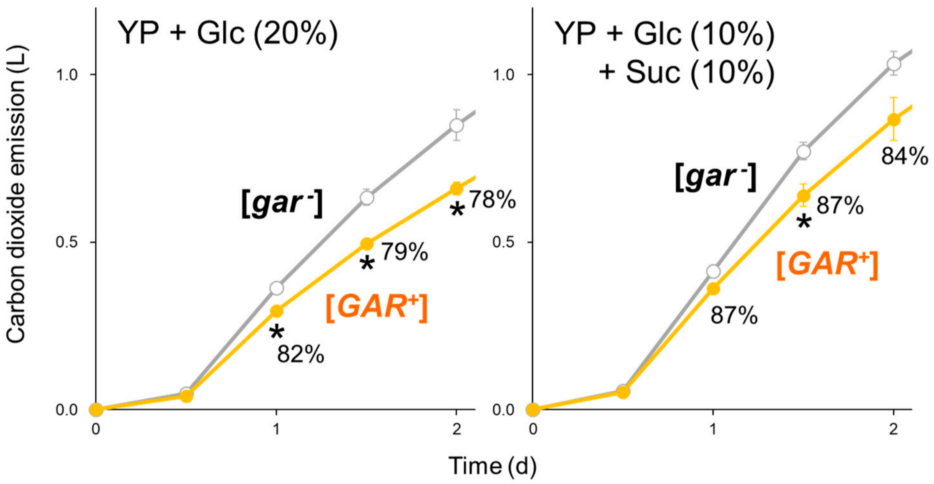

Supplementary Figure S1. [*GAR*$^+$] retards alcoholic fermentation when glucose is the sole carbon source. Carbon dioxide emission of [*gar*$^-$] (gray) and [*GAR*$^+$] (orange) strains in the X2180 background was monitored in YP + 20% glucose (left) or YP + 10% glucose + 10% sucrose (right) medium for 2 days. Data represent mean values ± standard deviations from three independent experiments. Percentages in the graph indicate how much emission was affected by [*GAR*$^+$]. Asterisks indicate that emission was statistically significantly decreased by [*GAR*$^+$]. (*t* test, $p < 0.05$).

Supplementary Figure S2 (Watanabe *et al.*)

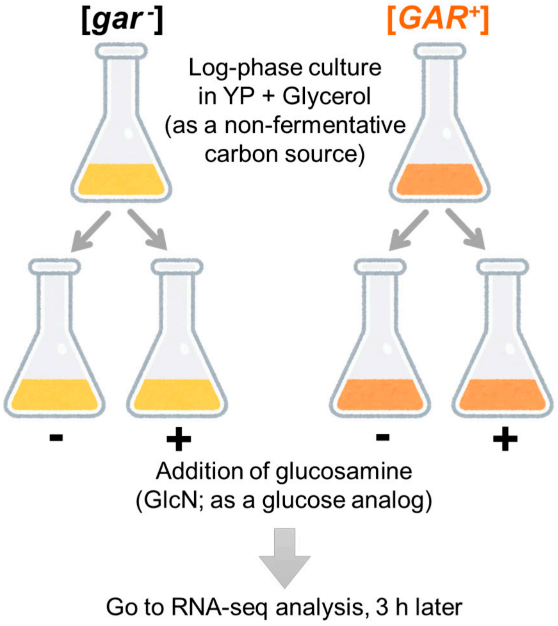

Supplementary Figure S2. RNA-seq to analyze the transcriptomic changes of the [*gar*$^-$] and [*GAR*$^+$] strains in response to glucosamine.

Supplementary Figure S3 (Watanabe *et al.*)

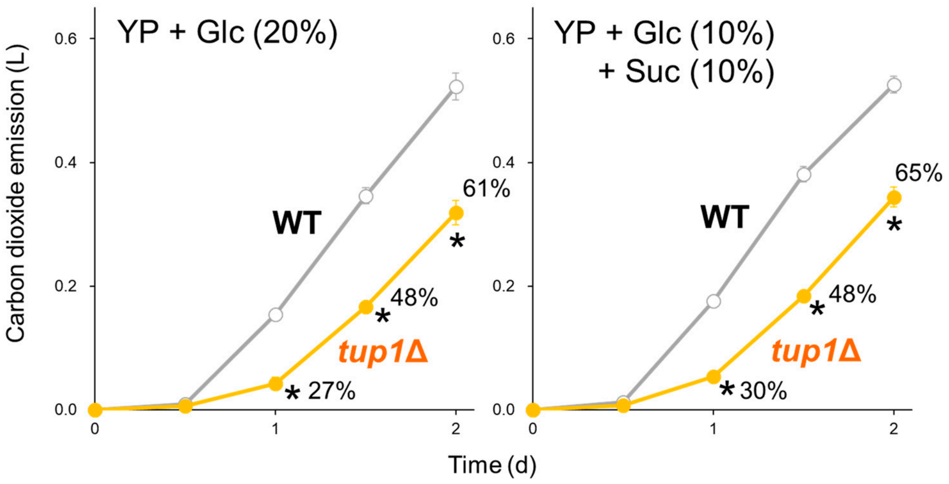

Supplementary Figure S3. Deletion of the *TUP1* gene retards alcoholic fermentation. Carbon dioxide emission of the wild type (WT; gray) and *tup1*Δ (orange) strains in the BY4741 background was monitored in YP + 20% glucose (left) or YP + 10% glucose + 10% sucrose (right) medium for 2 d. Data represent mean values ± standard deviations from three independent experiments. The percentages in the graph indicate how much emission was affected by *tup1*Δ. The asterisks indicate that emission significantly decreased by *tup1*Δ (*t* test, $p < 0.05$).

Supplementary Figure S4 (Watanabe *et al.*)

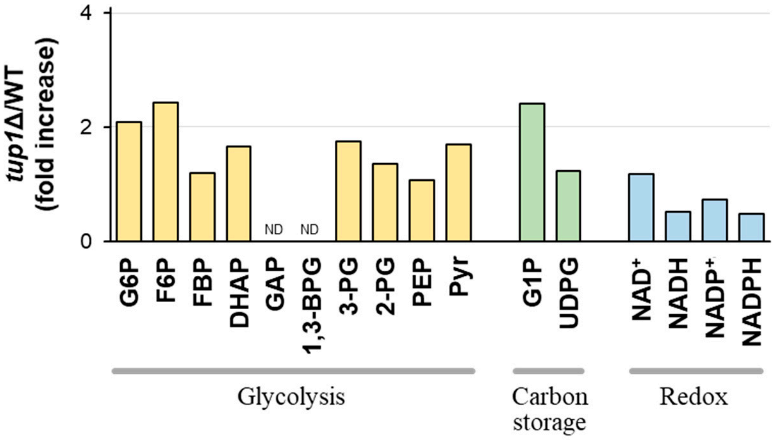

Supplementary Figure S4. Deletion of the *TUP1* gene affects carbon metabolism. Bar graphs represent fold increases in each metabolite level per cell compared to that in WT. G6P; glucose 6-phosphate. F6P; fructose 6-phosphate. FBP; fructose 1,6-bisphosphate. DHAP; dihydroxyacetone phosphate. GAP; glyceraldehyde 3- phosphate. 1,3-BPG; 1,3-bisphosphoglycerate. 3-PG; 3-phosphoglycerate. 2-PG; 2-phosphoglycerate. PEP; phosphoenolpyruvate. Pyr; pyruvate. AA; acetaldehyde. EtOH; ethanol. G1P; glucose 1-phosphate; UDPG; UDP-glucose. ND; not detected.

Supplementary Figure S5 (Watanabe *et al.*)

```
S288c  MNPGGEQTIMEQPAQQQQQQQQQQQQQQQQAAVPQQPLDPLTQSTAETWLSIASLAETLG 60
BY4741 ---------MEQPAQQQQQQQQQQQQQQQQAAVPQQPLDPLTQSTAETWLSIASLAETLG 51
X2180  MNPGGEQTIMEQPAQQQQQQQQQQQQQQQQAAVPQQPLDPLTQSTAETWLSIASLAETLG 60
K7     MNPGGEQTIMEQPAQQQQQQQQQQQQQQQQAAVPQQPLDPLTQSTAETWLSIASLAETLG 60
Km67   MNPGGEQTIMEQPAQQQQQQQQQQQ-----AAVPQQPLDPLTQSTAETWLSIASLAETLG 55
                ****************     ******************************

S288c  DGDRAAMAYDATLQFNPSSAKALTSLAHLYRSRDMFQRAAELYERALLVNPELSDVWATL 120
BY4741 DGDRAAMAYDATLQFNPSSAKALTSLAHLYRSRDMFQRAAELYERALLVNPELSDVWATL 111
X2180  DGDRAAMAYDATLQFNPSSAKALTSLAHLYRSRDMFQRAAELYERALLVNPELSDVWATL 120
K7     DGDRAAMAYDATLQFNPSSAKALTSLAHLYRSRDMFQRAAELYERALLVNPELSDVWVTL 120
Km67   DGDRAAMAYDATLQFNPSSAKALTSLAHLYRSRDMFQRAAELYERALLVNPELSDVWVTL 115
       *********************************************************.**

S288c  GHCYLMLDDLQRAYNAYQQALYHLSNPNVPKLWHGIGILYDRYGSLDYAEEAFAKVLELD 180
BY4741 GHCYLMLDDLQRAYNAYQQALYHLSNPNVPKLWHGIGILYDRYGSLDYAEEAFAKVLELD 171
X2180  GHCYLMLDDLQRAYNAYQQALYHLSNPNVPKLWHGIGILYDRYGSLDYAEEAFAKVLELD 180
K7     GHCYLMLDDLQRAYNAYQQALYHLSNPNVPKLWHGIGILYDRYGSLDYAEEAFAKVLELD 180
Km67   GHCYLMLDDLQRAYNAYQQALYHLSNPNVPKLWHGIGILYDRYGSLDYAEEAFAKVLELD 175
       ************************************************************

S288c  PHFEKANEIYFRLGIIYKHQGKWSQALECFRYILPQPPAPLQEWDIWFQLGSVLESMGEW 240
BY4741 PHFEKANEIYFRLGIIYKHQGKWSQALECFRYILPQPPAPLQEWDIWFQLGSVLESMGEW 231
X2180  PHFEKANEIYFRLGIIYKHQGKWSQALECFRYILPQPPAPLQEWDIWFQLGSVLESMGEW 240
K7     PHFEKTNEIYFRLGIIYKHQGKWSQALECFRYILPQPPAPLQEWDIWFQLGSVLESMGEW 240
Km67   PHFEKTNEIYFRLGIIYKHQGKWSQALECFRYILPQPPAPLQEWDIWFQLGSVLESMGEW 235
       *****:******************************************************

S288c  QGAKEAYEHVLAQNQHHAKVLQQLGCLYGMSNVQFYDPQKALDYLLKSLEADPSDATTWY 300
BY4741 QGAKEAYEHVLAQNQHHAKVLQQLGCLYGMSNVQFYDPQKALDYLLKSLEADPSDATTWY 291
X2180  QGAKEAYEHVLAQNQHHAKVLQQLGCLYGMSNVQFYDPQKALDYLLKSLEADPSDATTWY 300
K7     QGAKEAYEHVLAQNQHHAKVLQQLGCLYGMSNVQFYDPQKALDYLLKSLEADPSDATTWY 300
Km67   QGAKEAYEHVLAQNQHHAKVLQQLGCLYGMSNVQFYDPQKALDYLLKSLEADPSDATTWY 295
       ************************************************************

S288c  HLGRVHMIRTDYTAAYDAFQQAVNRDSRNPIFWCSIGVLYYQISQYRDALDAYTRAIRLN 360
BY4741 HLGRVHMIRTDYTAAYDAFQQAVNRDSRNPIFWCSIGVLYYQISQYRDALDAYTRAIRLN 351
X2180  HLGRVHMIRTDYTAAYDAFQQAVNRDSRNPIFWCSIGVLYYQISQYRDALDAYTRAIRLN 360
K7     HLGRVHMIRTDYTAAYDAFQQAVNRDSRNPIFWCSIGVLYYQISQYRDALDAYTRAIRLN 360
Km67   HLGRVHMIRTDYTAAYDAFQQAVNRDSRNPIFWCSIGVLYYQISQYRDALDAYTRAIRLN 355
       ************************************************************

S288c  PYISEVWYDLGTLYETCNNQLSDALDAYKQAARLDVNNVHIRERLEALTKQLENPGNINK 420
BY4741 PYISEVWYDLGTLYETCNNQLSDALDAYKQAARLDVNNVHIRERLEALTKQLENPGNINK 411
X2180  PYISEVWYDLGTLYETCNNQLSDALDAYKQAARLDVNNVHIRERLEALTKQLENPGNINK 420
K7     PYISEVWYDLGTLYETCNNQLSDALDAYKQAARLDVNNVHIRERLEALTKQLENPGNINK 420
Km67   PYISEVWYDLGTLYETCNNQLSDALDAYKQAARLDVNNVHIRERLEALTKQLENPGNINK 415
       ************************************************************

S288c  SNGAPTNASPAPPPVILQPTLQPNDQGNPLNTRISAQSANATASMVQQQHPAQQTPINSS 480
BY4741 SNGAPTNASPAPPPVILQPTLQPNDQGNPLNTRISAQSANATASMVQQQHPAQQTPINSS 471
X2180  SNGAPTNASPAPPPVILQPTLQPNDQGNPLNTRISAQSANATASMVQQQHPAQQTPINSS 480
K7     SNGAPTNASPAPPPVILQPTLQPNDQGNPLNTRISAQSANATASMVQQQHPAQQTPINSS 480
Km67   SNGAPTNASPAPPPVILQPTLQPNDQGNPLNTRISAQSANATASMVQQQHPAQQTPINSS 475
       ************************************************************
```

Supplementary Figure S5. Alignment of the amino acid sequences of the *CYC8* gene products of *S. cerevisiae* S288c, BY4741, X2180, K7, and Km67 strains. The sequences of S288c, BY4741, X2180, and K7 were obtained from the *Saccharomyces* Genome Database (SGD; https://www.yeastgenome.org/). Red letters indicate mutated amino acids only in Km67. Orange letters indicate mutated amino acids commonly in sake strains (K7 and Km67).

Supplementary Figure S5 (Watanabe *et al.*)

```
S288c  ATMYSNGASPQLQAQAQAQAQAQAQAQAQAQAQAQAQAQAQAQAQAQAQAQAQAQA---- 536
BY4741 ATMYSNGASPQLQAQAQAQAQAQAQAQAQAQAQAQAQAQAQAQAQAQAQAQAQAQA---- 527
X2180  ATMYSNGASPQLQAQAQAQAQAQAQAQAQAQAQAQAQAQAQAQAQAQAQAQAQAQA---- 536
K7     ATMYSNGASPQLQAQAQAQAQAQAQAQAQAQAQAQAQAQAQAQAQAQAQAQAQAQAQAQA 540
Km67   ATMYSNGASPQLQAQAQAQAQAQAQAQAQAQAQAQAQAQAQAQAQAQAQAQAQAQAQAQA 535
       ********************************************************

S288c  HAQAQAQAQAQAQAQAQAQAQQQQQQQQQQQQQQQQQQQQQQQQQ--------QQQQQQL 588
BY4741 HAQAQAQAQAQAQAQAQAQAQQQQQQQQQQQQQQQQQQQQQQQQQQQQQQQQQQQQQQQL 587
X2180  HAQAQAQAQAQAQAQAQAQAQQQQQQQQQQQQQQQQQQQQQQQ----------QQQQQQL 586
K7     HAQAQAQAQAQAQAQAQAQAQQQQQQQQQQQQQQQQQQQQQQQQQQK------QQQQQQL 594
Km67   HAQAQAQAQAQAQAQAQAQAQQQQQQQQRQQQQQQQQQQQQRQQQQK------QQQQQQL 589
       ****************************:************:***        *******

S288c  QPLPRQQLQQKGVSVQMLNPQQGQPYITQPTVIQAHQLQPFSTQAMEHPQSSQLPPQQQQ 648
BY4741 QPLPRQQLQQKGVSVQMLNPQQGQPYITQPTVIQAHQLQPFSTQAMEHPQSSQLPPQQQQ 647
X2180  QPLPRQQLQQKGVSVQMLNPQQGQPYITQPTVIQAHQLQPFSTQAMEHPQSSQLPPQQQQ 646
K7     QPLPRQQLQQKGVSVQMLNPQQGQPYITQPIVIQAHQLQPFSTQAMEHPQSSQLPPQQQQ 654
Km67   QPLPRQQLQQKGVSVQMLNPQQGQPYITQPIVIQAHQLQPFSTQAMEHPQSSQLPPQQQQ 649
       ****************************** *****************************

S288c  LQSVQHPQQLQGQPQAQAPQPLIQHNVEQNVLPQKRYMEGAIHTLVDAAVSSSTHTENNT 708
BY4741 LQSVQHPQQLQGQPQAQAPQPLIQHNVEQNVLPQKRYMEGAIHTLVDAAVSSSTHTENNT 707
X2180  LQSVQHPQQLQGQPQAQAPQPLIQHNVEQNVLPQKRYMEGAIHTLVDAAVSSSTHTENNT 706
K7     LQSVQHPQQLQGQPQAQAPQPLIQHNVEQNVLPQKRYMEGAIHTLVDAAVSSSTHTENNT 714
Km67   LQSVQHPQQLQGQPQAQAPQPLIQHNVEQNVLPQKRYMEGAIHTLVDAAVSSSTHTENNT 709
       ************************************************************

S288c  KSPRQPTHAIPTQAPATGITNAEPQVKKQKLNSPNSNINKLVNTATSIEENAKSEVSNQS 768
BY4741 KSPRQPTHAIPTQAPATGITNAEPQVKKQKLNSPNSNINKLVNTATSIEENAKSEVSNQS 767
X2180  KSPRQPTHAIPTQAPATGITNAEPQVKKQKLNSPNSNINKLVNTATSIEENAKSEVSNQS 766
K7     KSPRQPTHAIPTQAPATGITNAEPQVKKQKLNSPNSNINKLVNTATSIEENAKSEVSNQS 774
Km67   KSPRQPTHAIPTQAPATGITNAEPRVKKQKLNSPNSNINKLVNTATSIEENAKSEVSNQS 769
       ************************:***********************************

S288c  PAVVESNTNNTSQEEKPVKANSIPSVIGAQEPPQEASPAEEATKAASVSPSTKPLNTEPE 828
BY4741 PAVVESNTNNTSQEEKPVKANSIPSVIGAQEPPQEASPAEEATKAASVSPSTKPLNTEPE 827
X2180  PAVVESNTNNTSQEEKPVKANSIPSVIGAQEPPQEASPAEEATKAASVSPSTKPLNTEPE 826
K7     PAVVESNTNNTSQEEKPVKANSIPSVIGAQEPPQEASPAEEATKAASVSPSTKPLNTEPE 834
Km67   PAVVESNTNNTSQEEKPVKANSIPSVIGAQEPPQEASPAEEATKAASVSPSTKPLNTEPE 829
       ************************************************************

S288c  SSSVQPTVSSESSTTKANDQSTAETIELSTATVPAEASPVEDEVRQHSKEENGTTEASAP 888
BY4741 SSSVQPTVSSESSTTKANDQSTAETIELSTATVPAEASPVEDEVRQHSKEENGTTEASAP 887
X2180  SSSVQPTVSSESSTTKANDQSTAETIELSTATVPAEASPVEDEVRQHSKEENGTTEASAP 886
K7     SSSVQPTVSSESSTTKANDQSTAETIELSTATVPAEASPVEDEVRQHSKEENGTTEASAP 894
Km67   SSSVQPTVSSESSTTKANDQSTAETIELSTATVPAEASPVEDEVRQHSKEENGTTEASAP 889
       ************************************************************

S288c  STEEAEPAASRDAEKQQDETAATTITVIKPTLETMETVKEEAKMREEEQTSQEKSPQENT 948
BY4741 STEEAEPAASRDAEKQQDETAATTITVIKPTLETMETVKEEAKMREEEQTSQEKSPQENT 947
X2180  STEEAEPAASRDAEKQQDETAATTITVIKPTLETMETVKEEAKMREEEQTSQEKSPQENT 946
K7     STEEAEPAASRDAEKQQDETAATTITVIKPTLETMETVKEEAKMREEEQTSQEKSPQENT 954
Km67   STEEAEPAASRDAEKQQDETAATTITVIKPTLETMETVKEEAKMREEEQTSQEKSPQENT 949
       ************************************************************

S288c  LPRENVVRQVEEDENYDD 966
BY4741 LPRENVVRQVEEDENYDD 965
X2180  LPRENVVRQVEEDENYDD 964
K7     LPRENVVRQVEEDENYDD 972
Km67   LPRENVVRQVEEDENYDD 967
       ******************
```

Supplementary Figure S5. (continued)

Supplementary Table S1. Genes with increased induction ratios in [*GAR+*] cells (> 2).

Gene ID	Name	[*gar-*] /- GlcN (A)	[*gar-*] /+ GlcN (B)	[*GAR+*] /- GlcN (C)	[*GAR+*] /+ GlcN (D)	Fold change (C/A)	Fold change (D/B)	Induction ratio (D/C)/(B/A)
YAL040C	*'CLN3*	127.5	32.0	45.8	28.5	0.359	0.891	2.48
YAL034W-A	*'MTW1*	34.4	12.8	14.8	13.0	0.431	1.021	2.37
YBL002W	*'HTB2*	2939.7	1182.9	1589.0	1323.8	0.541	1.119	2.07
YBL003C	*'HTA2*	3344.0	2052.4	1868.8	2348.8	0.559	1.144	2.05
YBR050C	*'REG2*	88.1	39.9	49.8	47.3	0.565	1.185	2.10
YER067W	*'RGI1*	209.3	19.3	86.9	23.8	0.415	1.232	2.97
YPL057C	*'SUR1*	502.4	77.1	242.5	97.0	0.483	1.258	2.61
YPR192W	*'AQY1*	24.8	84.5	15.1	108.9	0.610	1.289	2.11
YBR145W	*'ADH5*	74.6	119.7	39.9	159.1	0.535	1.329	2.48
YGL055W	*'OLE1*	4127.2	125.2	2511.3	167.4	0.608	1.337	2.20
YLR377C	*'FBP1*	123.7	26.9	56.2	36.3	0.454	1.348	2.97
YDR076W	*'RAD55*	38.2	23.1	25.2	31.6	0.659	1.368	2.08
YDR536W	*'STL1*	142.3	15.8	43.5	21.9	0.306	1.380	4.51
YDR360W	*'OPI7*	18.5	15.1	11.5	20.8	0.623	1.381	2.22
YLR023C	*'IZH3*	361.1	196.4	228.2	278.1	0.632	1.416	2.24
YOR081C	*'TGL5*	35.9	18.7	23.3	26.5	0.649	1.421	2.19
YGL089C	*'MF(ALPHA)2*	33.2	20.3	18.3	29.7	0.551	1.462	2.65
YAL033W	*'POP5*	101.6	40.7	61.6	61.0	0.606	1.497	2.47
YEL061C	*'CIN8*	33.5	11.8	22.6	17.9	0.674	1.510	2.24
YLR315W	*'NKP2*	40.6	19.1	24.2	29.0	0.598	1.522	2.55
YBR030W	*'RKM3*	60.6	23.4	42.0	36.0	0.693	1.536	2.21
YMR316W	*'DIA1*	64.9	71.2	46.2	110.4	0.712	1.550	2.18
YNL117W	*'MLS1*	29.2	19.9	17.8	31.1	0.610	1.568	2.57
YIL150C	*'MCM10*	19.4	12.3	13.2	19.5	0.680	1.582	2.33
YJL116C	*'NCA3*	457.5	510.8	320.2	809.0	0.700	1.584	2.26
YOR380W	*'RDR1*	17.5	10.9	12.9	17.3	0.738	1.592	2.16
YDL171C	*'GLT1*	41.2	105.6	30.4	168.4	0.739	1.596	2.16
YJL019W	*'MPS3*	22.0	13.8	17.5	22.1	0.793	1.607	2.03
YGR031W	*'IMO32*	106.0	43.4	70.4	69.9	0.664	1.610	2.43
YJL090C	*'DPB11*	38.5	15.9	30.5	25.7	0.793	1.611	2.03
YHL039W	*'EFM1*	143.4	38.3	84.6	62.0	0.590	1.619	2.74
YGR031C-A	*'NAG1*	143.7	58.0	110.7	94.1	0.770	1.621	2.10
YDR019C	*'GCV1*	210.8	42.0	167.2	68.3	0.793	1.625	2.05
YDR123C	*'INO2*	46.9	20.1	35.8	33.1	0.763	1.644	2.16
YOL017W	*'ESC8*	23.0	14.3	18.7	23.5	0.814	1.648	2.02
YGL056C	*'SDS23*	361.9	100.5	274.7	165.9	0.759	1.650	2.17
YKL143W	*'LTV1*	162.3	43.0	129.9	71.2	0.800	1.656	2.07
YPL096C-A	*'ERI1*	82.8	134.1	55.4	226.1	0.669	1.686	2.52
YLR432W	*'IMD3*	398.0	233.3	333.7	394.0	0.838	1.689	2.01
YDL240W	*'LRG1*	31.5	10.4	23.9	17.9	0.758	1.721	2.27
YER118C	*'SHO1*	72.2	25.9	61.8	44.6	0.857	1.721	2.01
YPL226W	*'NEW1*	312.9	53.0	250.5	91.4	0.801	1.723	2.15
YBR004C	*'GPI18*	98.1	41.2	74.6	72.1	0.761	1.751	2.30
YGL162W	*'SUT1*	27.4	13.0	20.8	22.9	0.758	1.766	2.33
YNL112W	*'DBP2*	1424.5	104.3	817.5	184.3	0.574	1.767	3.08
YNL187W	*'SWT21*	41.2	30.3	32.5	53.6	0.788	1.770	2.25
YML017W	*'PSP2*	54.2	22.9	45.9	40.6	0.847	1.774	2.09
YBR233W-A	*'DAD3*	241.5	138.0	212.3	246.4	0.879	1.785	2.03
YIL026C	*'IRR1*	24.5	11.3	20.0	20.2	0.814	1.788	2.20
YGL254W	*'FZF1*	59.8	25.5	48.4	45.7	0.809	1.790	2.21
YER032W	*'FIR1*	30.2	10.2	26.9	18.4	0.890	1.803	2.02
YML120C	*'NDI1*	509.4	228.9	360.8	415.4	0.708	1.815	2.56
YDL055C	*'PSA1*	1385.4	211.1	1141.9	383.8	0.824	1.819	2.21
YJL187C	*'SWE1*	39.2	17.9	30.0	32.6	0.765	1.822	2.38
YDR097C	*'MSH6*	30.4	12.1	26.0	22.2	0.853	1.828	2.14
YER065C	*'ICL1*	116.3	81.3	71.3	148.8	0.613	1.830	2.98
YML087C	*'AIM33*	170.0	58.6	155.5	107.7	0.915	1.837	2.01
YKR097W	*'PCK1*	69.5	10.2	31.7	18.8	0.456	1.849	4.06
YPL241C	*'CIN2*	41.8	26.6	33.2	49.2	0.793	1.850	2.33
YOR206W	*'NOC2*	214.8	51.5	187.9	95.4	0.875	1.853	2.12

YML085C	*'TUB1*	302.6	67.5	270.1	125.2	0.893	1.855	2.08
YOR073W	*'SGO1*	26.4	10.6	22.8	19.8	0.865	1.862	2.15
YNL313C	*'EMW1*	82.5	21.2	76.0	40.0	0.922	1.889	2.05
YLR342W	*'FKS1*	212.3	38.0	160.4	72.3	0.756	1.900	2.52
YPL126W	*'NAN1*	159.1	25.5	151.3	49.1	0.951	1.925	2.02
YCR072C	*'RSA4*	136.4	31.5	121.3	61.1	0.889	1.940	2.18
YPL093W	*'NOG1*	394.2	71.2	387.1	140.9	0.982	1.979	2.02
YPR190C	*'RPC82*	128.5	48.2	111.3	95.7	0.866	1.984	2.29
YPR159W	*'KRE6*	138.7	66.0	137.4	131.0	0.990	1.985	2.01
YMR290C	*'HAS1*	249.7	71.6	234.2	142.3	0.938	1.987	2.12
YER003C	*'PMI40*	199.5	73.6	189.4	147.4	0.949	2.002	2.11
YPL183C	*'RTT10*	69.2	11.4	64.5	23.0	0.932	2.017	2.16
YDL198C	*'GGC1*	219.2	41.5	193.9	84.4	0.885	2.030	2.29
YNL283C	*'WSC2*	85.9	21.5	68.0	43.8	0.791	2.036	2.57
YBR112C	*'CYC8*	81.7	12.4	73.8	25.4	0.904	2.037	2.25
YDR021W	*'FAL1*	61.7	17.4	54.4	35.7	0.881	2.052	2.33
YGL038C	*'OCH1*	113.6	43.2	95.9	88.8	0.844	2.055	2.43
YLR222C	*'UTP13*	114.0	21.7	100.6	44.6	0.883	2.059	2.33
YML056C	*'IMD4*	432.4	112.2	340.9	231.3	0.788	2.061	2.61
YDL010W	*'GRX6*	171.1	52.2	135.6	107.7	0.792	2.062	2.60
YLR451W	*'LEU3*	36.1	10.6	33.1	21.8	0.915	2.062	2.25
YOR315W	*'SFG1*	141.4	15.2	143.0	31.5	1.011	2.063	2.04
YNL268W	*'LYP1*	364.6	128.0	358.0	264.3	0.982	2.065	2.10
YDR437W	*'GPI19*	26.8	11.4	21.5	23.7	0.804	2.070	2.57
YJR132W	*'NMD5*	28.9	26.1	27.6	54.1	0.957	2.075	2.17
YHR196W	*'UTP9*	175.1	40.2	172.4	83.6	0.985	2.082	2.11
YLR174W	*'IDP2*	365.9	88.1	258.7	184.3	0.707	2.091	2.96
YJR143C	*'PMT4*	111.1	25.3	104.0	53.1	0.936	2.097	2.24
YNL298W	*'CLA4*	40.7	14.9	39.7	31.3	0.975	2.099	2.15
YML027W	*'YOX1*	149.6	40.7	105.9	85.4	0.708	2.100	2.97
YCR057C	*'PWP2*	130.1	25.0	113.7	52.5	0.874	2.101	2.40
YKL217W	*'JEN1*	1656.6	88.4	1199.1	187.4	0.724	2.120	2.93
YDL063C	*'SYO1*	76.9	17.9	55.6	38.1	0.722	2.123	2.94
YOR033C	*'EXO1*	30.1	12.5	30.0	26.6	0.997	2.129	2.14
YJL050W	*'MTR4*	106.8	14.5	84.7	30.9	0.793	2.136	2.70
YKR099W	*'BAS1*	80.7	14.9	71.3	31.9	0.884	2.137	2.42
YCL036W	*'GFD2*	108.4	19.1	107.1	40.8	0.988	2.137	2.16
YGR243W	*'MPC3*	781.6	76.0	629.4	163.5	0.805	2.153	2.67
YLR168C	*'UPS2*	435.6	321.1	436.7	692.4	1.003	2.156	2.15
YKL148C	*'SDH1*	377.7	170.4	345.9	371.6	0.916	2.181	2.38
YJL085W	*'EXO70*	32.1	13.7	30.9	30.0	0.964	2.190	2.27
YBL039C	*'URA7*	255.9	54.2	255.8	118.8	0.999	2.191	2.19
YPL156C	*'PRM4*	215.6	158.3	210.6	347.2	0.977	2.193	2.25
YBR029C	*'CDS1*	154.8	55.2	139.5	121.2	0.901	2.196	2.44
YLR426W	*'TDA5*	58.5	35.9	58.8	79.0	1.005	2.200	2.19
YBL042C	*'FUI1*	149.9	15.3	71.6	33.6	0.478	2.203	4.61
YGR183C	*'QCR9*	4447.0	1661.7	3690.6	3662.2	0.830	2.204	2.66
YDL167C	*'NRP1*	89.2	33.7	75.2	74.6	0.843	2.213	2.63
YLR249W	*'YEF3*	2264.1	423.8	1988.2	938.5	0.878	2.214	2.52
YER110C	*'KAP123*	135.6	30.8	131.3	68.8	0.968	2.235	2.31
YMR095C	*'SNO1*	29.6	58.6	32.4	131.6	1.097	2.245	2.05
YLL008W	*'DRS1*	204.4	57.6	185.6	129.5	0.908	2.248	2.48
YOL011W	*'PLB3*	89.3	34.3	66.9	77.3	0.750	2.251	3.00
YBR142W	*'MAK5*	87.7	15.2	75.0	34.4	0.856	2.266	2.65
YCR065W	*'HCM1*	61.3	15.2	51.3	34.6	0.837	2.272	2.72
YER064C	*'VHR2*	213.5	67.0	170.7	152.9	0.800	2.282	2.85
YML091C	*'RPM2*	182.2	64.3	183.3	146.9	1.006	2.285	2.27
YLL041C	*'SDH2*	590.2	314.0	470.1	718.3	0.797	2.288	2.87
YDL148C	*'NOP14*	128.8	31.8	105.3	72.9	0.818	2.291	2.80
YMR309C	*'NIP1*	234.2	42.3	232.8	97.5	0.994	2.304	2.32
YMR307W	*'GAS1*	1151.6	220.5	1050.3	513.6	0.912	2.330	2.55
YLR212C	*'TUB4*	54.0	19.5	49.9	45.6	0.923	2.330	2.53
YIL131C	*'FKH1*	50.5	11.6	49.5	27.1	0.980	2.332	2.38
YOR375C	*'GDH1*	624.7	322.8	709.0	753.2	1.135	2.333	2.06
YPL127C	*'HHO1*	403.0	65.4	341.2	153.5	0.847	2.347	2.77

YLR002C	'NOC3	102.1	24.3	99.3	57.5	0.973	2.366	2.43
YHR197W	'RIX1	65.7	12.0	55.9	28.6	0.850	2.381	2.80
YML026C	'RPS18B	3475.1	345.4	4006.6	824.4	1.153	2.387	2.07
YDR324C	'UTP4	126.5	21.5	123.0	51.6	0.972	2.401	2.47
YBL015W	'ACH1	368.4	85.0	280.4	206.7	0.761	2.431	3.19
YMR049C	'ERB1	143.3	22.5	124.3	54.8	0.867	2.434	2.81
YMR177W	'MMT1	31.7	12.9	38.2	31.4	1.203	2.436	2.02
YGR128C	'UTP8	92.6	17.5	74.8	42.6	0.808	2.439	3.02
YLR413W	'INA1	230.3	24.6	168.5	60.2	0.732	2.444	3.34
YMR229C	'RRP5	90.3	14.0	74.5	34.4	0.826	2.457	2.98
YLR314C	'CDC3	196.8	54.8	236.3	135.3	1.201	2.470	2.06
YKL101W	'HSL1	37.0	10.5	33.5	26.3	0.905	2.499	2.76
YDR020C	'DAS2	49.1	14.9	49.4	37.3	1.008	2.501	2.48
YDR044W	'HEM13	59.8	12.9	69.4	32.3	1.161	2.504	2.16
YBR070C	'ALG14	108.6	21.5	90.1	53.9	0.830	2.506	3.02
YHR010W	'RPL27A	4082.3	517.0	5121.7	1298.4	1.255	2.511	2.00
YGL061C	'DUO1	51.3	16.9	45.0	42.7	0.878	2.529	2.88
YDR354W	'TRP4	103.9	42.5	128.8	108.1	1.240	2.545	2.05
YPR051W	'MAK3	228.4	60.2	280.6	154.2	1.229	2.563	2.09
YLR376C	'PSY3	49.9	16.5	54.5	42.3	1.091	2.566	2.35
YCR063W	'BUD31	33.2	11.7	35.9	30.0	1.080	2.570	2.38
YGR279C	'SCW4	743.7	75.8	851.3	195.3	1.145	2.578	2.25
YDR528W	'HLR1	53.6	12.2	55.7	31.8	1.039	2.605	2.51
YOL105C	'WSC3	44.5	23.9	48.8	62.5	1.098	2.611	2.38
YOL123W	'HRP1	287.0	69.0	368.9	180.5	1.286	2.614	2.03
YNR003C	'RPC34	100.2	37.2	120.5	97.3	1.203	2.615	2.17
YGR189C	'CRH1	367.0	162.1	371.9	427.7	1.013	2.638	2.60
YGL031C	'RPL24A	3319.6	245.8	4062.4	649.4	1.224	2.641	2.16
YKR013W	'PRY2	329.1	100.2	239.9	267.4	0.729	2.670	3.66
YJL198W	'PHO90	83.9	11.2	71.5	30.0	0.852	2.670	3.13
YBR069C	'TAT1	586.7	129.4	560.7	348.6	0.956	2.693	2.82
YIL123W	'SIM1	191.1	37.0	177.3	99.8	0.928	2.694	2.90
YLR154C	'RNH203	139.2	63.0	140.3	170.9	1.008	2.715	2.69
YMR128W	'ECM16	65.8	11.1	57.8	30.1	0.878	2.718	3.09
YBR298C	'MAL31	140.8	20.7	119.8	56.9	0.851	2.742	3.22
YJL102W	'MEF2	43.5	12.7	49.4	34.8	1.134	2.742	2.42
YBL030C	'PET9	2174.2	321.3	2111.3	882.8	0.971	2.748	2.83
YNL132W	'KRE33	71.5	12.3	68.4	34.0	0.957	2.756	2.88
YDR384C	'ATO3	231.6	79.7	275.1	220.7	1.188	2.767	2.33
YDL052C	'SLC1	226.9	65.9	258.1	187.4	1.137	2.844	2.50
YGR295C	'COS6	250.1	71.8	315.9	204.5	1.263	2.846	2.25
YCR034W	'ELO2	220.5	34.8	267.6	99.8	1.214	2.871	2.37
YDL101C	'DUN1	24.1	10.1	28.8	29.0	1.196	2.884	2.41
YGL077C	'HNM1	119.1	32.1	140.7	92.9	1.181	2.897	2.45
YDR060W	'MAK21	145.8	20.7	132.2	60.4	0.907	2.913	3.21
YMR003W	'AIM34	90.7	23.7	95.3	69.5	1.051	2.929	2.79
YER024W	'YAT2	23.2	70.7	31.3	209.4	1.346	2.964	2.20
YGL076C	'RPL7A	2009.4	268.3	2614.3	795.6	1.301	2.966	2.28
YOR247W	'SRL1	464.5	44.2	414.0	134.4	0.891	3.037	3.41
YMR215W	'GAS3	89.5	11.7	79.2	35.8	0.885	3.061	3.46
YER095W	'RAD51	132.9	52.0	148.7	160.3	1.119	3.080	2.75
YBR297W	'MAL33	177.4	27.3	193.2	84.2	1.089	3.081	2.83
YKR077W	'MSA2	59.6	17.4	57.6	54.6	0.967	3.135	3.24
YPL267W	'ACM1	89.3	17.7	88.3	56.4	0.989	3.181	3.22
YDR144C	'MKC7	35.9	11.3	54.9	36.1	1.529	3.187	2.08
YBR084W	'MIS1	125.2	10.4	128.4	33.2	1.025	3.200	3.12
YPL012W	'RRP12	142.3	10.6	120.7	35.5	0.848	3.344	3.94
YGL253W	'HXK2	748.0	196.0	1132.6	672.7	1.514	3.433	2.27
YLR372W	'ELO3	271.4	38.7	327.2	133.8	1.206	3.461	2.87
YOR108W	'LEU9	87.7	22.8	103.0	80.6	1.174	3.543	3.02
YCL026C-B	'HBN1	60.1	19.5	89.7	69.5	1.493	3.566	2.39
YGR103W	'NOP7	314.8	42.0	366.9	151.5	1.165	3.606	3.09
YPR010C	'RPA135	170.9	15.4	153.8	55.7	0.900	3.622	4.03
YNR009W	'NRM1	71.7	11.1	99.3	40.3	1.383	3.626	2.62
YPR002W	'PDH1	89.9	72.5	140.2	264.4	1.559	3.646	2.34

YCR010C	*'ADY2*	39.9	21.5	28.8	78.7	0.721	3.666	5.09
YEL040W	*'UTR2*	209.7	52.1	262.8	199.8	1.253	3.839	3.06
YMR199W	*'CLN1*	198.7	13.8	112.9	54.0	0.568	3.915	6.89
YBR021W	*'FUR4*	49.5	16.6	78.0	69.2	1.576	4.174	2.65
YDR309C	*'GIC2*	59.1	17.3	67.4	82.2	1.140	4.744	4.16
YOR100C	*'CRC1*	29.0	32.9	37.7	158.3	1.301	4.807	3.70
YOL007C	*'CSI2*	97.0	12.8	74.0	62.7	0.763	4.915	6.44
YJR095W	*'SFC1*	116.8	12.3	124.6	71.3	1.066	5.810	5.45
YMR011W	*'HXT2*	1143.7	79.6	3115.9	465.6	2.724	5.851	2.15
YNL301C	*'RPL18B*	445.3	47.2	728.0	293.8	1.635	6.223	3.81
YMR305C	*'SCW10*	296.8	26.3	209.5	201.8	0.706	7.688	10.89
YNL300W	*'TOS6*	263.8	21.6	258.4	177.1	0.979	8.187	8.36
YPL163C	*'SVS1*	223.8	10.8	182.2	114.8	0.814	10.628	13.06

Supplementary Table S2. Genes with decreased induction ratios in [*GAR+*] cells (< 0.5).

Gene ID	Name	[*gar-*] /- GlcN (A)	[*gar-*] /+ GlcN (B)	[*GAR+*] /- GlcN (C)	[*GAR+*] /+ GlcN (D)	Fold change (C/A)	Fold change (D/B)	Induction ratio (D/C)/(B/A)
YPL223C	*'GRE1*	297.1	3521.2	36.6	183.2	0.123	0.052	0.42
YMR175W	*'SIP18*	10528.0	17046.6	2211.5	992.3	0.210	0.058	0.28
YGR256W	*'GND2*	107.6	476.8	40.8	31.7	0.380	0.066	0.17
YMR169C	*'ALD3*	94.4	659.5	44.1	52.8	0.467	0.080	0.17
YMR174C	*'PAI3*	6149.8	23619.7	1717.0	1939.6	0.279	0.082	0.29
YAL061W	*'BDH2*	169.3	456.0	73.7	42.2	0.435	0.093	0.21
YDL222C	*'FMP45*	85.3	1112.4	60.0	114.6	0.703	0.103	0.15
YDR070C	*'FMP16*	478.7	2735.9	252.0	290.8	0.526	0.106	0.20
YDL204W	*'RTN2*	460.1	1168.3	242.7	136.5	0.528	0.117	0.22
YGR088W	*'CTT1*	23.2	626.4	15.0	74.1	0.646	0.118	0.18
YML128C	*'MSC1*	754.7	2540.2	352.1	308.3	0.467	0.121	0.26
YIL136W	*'OM45*	1116.2	13413.8	627.5	1690.4	0.562	0.126	0.22
YDR453C	*'TSA2*	151.7	335.9	79.7	47.7	0.525	0.142	0.27
YCR021C	*'HSP30*	37.4	1104.3	20.4	157.4	0.546	0.143	0.26
YBR117C	*'TKL2*	138.3	931.2	39.3	133.0	0.284	0.143	0.50
YMR250W	*'GAD1*	193.5	1996.4	129.8	286.8	0.671	0.144	0.21
YBL075C	*'SSA3*	56.1	1010.6	35.4	154.1	0.631	0.152	0.24
YER150W	*'SPI1*	289.1	4935.9	93.0	755.3	0.322	0.153	0.48
YOL052C-A	*'DDR2*	5763.9	27091.7	2782.2	4559.3	0.483	0.168	0.35
YHR096C	*'HXT5*	211.9	1238.4	108.3	210.6	0.511	0.170	0.33
YGR043C	*'NQM1*	252.2	1026.6	116.0	176.4	0.460	0.172	0.37
YOR382W	*'FIT2*	24184.0	4020.3	18979.5	721.9	0.785	0.180	0.23
YNL160W	*'YGP1*	1513.9	4564.8	884.3	892.1	0.584	0.195	0.33
YDL223C	*'HBT1*	85.1	153.1	42.1	32.5	0.495	0.212	0.43
YGR248W	*'SOL4*	47.3	571.2	52.1	121.4	1.101	0.212	0.19
YOR161C	*'PNS1*	67.7	306.2	60.0	66.0	0.886	0.215	0.24
YAL055W	*'PEX22*	179.0	277.9	81.3	60.7	0.454	0.219	0.48
YPL186C	*'UIP4*	404.4	304.7	194.1	67.4	0.480	0.221	0.46
YOR383C	*'FIT3*	22526.5	4712.8	16963.0	1078.8	0.753	0.229	0.30
YMR195W	*'ICY1*	147.9	1344.4	129.2	313.4	0.874	0.233	0.27
YIR038C	*'GTT1*	676.5	1381.5	415.6	330.1	0.614	0.239	0.39
YGR236C	*'SPG1*	17.4	153.0	17.6	36.6	1.012	0.239	0.24
YAR071W	*'PHO11*	16.5	151.3	30.5	36.2	1.846	0.239	0.13
YPR101W	*'SNT309*	164.8	1852.8	166.4	456.7	1.010	0.247	0.24
YDR276C	*'PMP3*	6382.1	24336.6	3703.6	6023.0	0.580	0.247	0.43
YNL015W	*'PBI2*	5265.1	6410.2	4162.0	1671.4	0.790	0.261	0.33
YIL101C	*'XBP1*	43.3	116.4	41.2	31.2	0.953	0.268	0.28
YPR160W	*'GPH1*	216.3	231.5	183.4	63.0	0.848	0.272	0.32
YEL039C	*'CYC7*	76.9	1025.9	142.5	280.5	1.852	0.273	0.15
YPL076W	*'GPI2*	32.7	326.5	25.9	90.2	0.793	0.276	0.35
YPR149W	*'NCE102*	1277.6	4543.4	1655.9	1283.2	1.296	0.282	0.22
YLR258W	*'GSY2*	198.1	654.0	159.6	188.0	0.805	0.287	0.36
YOL151W	*'GRE2*	123.1	268.8	106.5	78.5	0.865	0.292	0.34
YMR105C	*'PGM2*	284.5	358.2	219.8	105.1	0.773	0.293	0.38
YLR119W	*'SRN2*	58.8	391.2	47.7	115.5	0.810	0.295	0.36
YDR281C	*'PHM6*	151.5	2389.7	141.7	709.0	0.935	0.297	0.32
YOR257W	*'CDC31*	296.7	1610.1	231.7	482.3	0.781	0.300	0.38
YER103W	*'SSA4*	84.8	1496.6	58.3	448.5	0.688	0.300	0.44
YMR118C	*'SHH3*	31.3	176.1	32.2	53.2	1.029	0.302	0.29
YHR087W	*'RTC3*	193.7	374.3	135.4	113.6	0.699	0.304	0.43
YML100W	*'TSL1*	69.2	394.0	72.3	122.4	1.043	0.311	0.30
YGR008C	*'STF2*	2878.9	4145.0	1818.2	1293.7	0.632	0.312	0.49
YPL250C	*'ICY2*	64.3	489.8	67.4	155.8	1.048	0.318	0.30
YPL222W	*'FMP40*	60.6	277.7	41.6	91.3	0.687	0.329	0.48
YOL155C	*'HPF1*	2610.3	411.8	2335.5	138.1	0.895	0.335	0.37
YJR086W	*'STE18*	28.7	36.1	28.1	12.1	0.978	0.336	0.34
YMR255W	*'GFD1*	253.1	1483.1	239.6	499.0	0.947	0.336	0.36

YDR253C	'MET32	53.5	182.1	61.2	64.3	1.144	0.353	0.31
YPL004C	'LSP1	1188.0	2132.3	993.2	754.1	0.836	0.354	0.42
YBR093C	'PHO5	128.9	965.0	638.7	342.3	4.955	0.355	0.07
YJR155W	'AAD10	60.2	1228.4	52.6	437.8	0.874	0.356	0.41
YAL038W	'CDC19	671.1	2932.7	500.1	1065.9	0.745	0.363	0.49
YJR073C	'OPI3	954.9	1629.4	918.4	592.6	0.962	0.364	0.38
YBR054W	'YRO2	166.2	3715.4	241.2	1357.9	1.451	0.365	0.25
YER081W	'SER3	76.8	387.9	101.6	142.1	1.324	0.366	0.28
YNL136W	'EAF7	111.9	529.8	107.5	196.7	0.961	0.371	0.39
YMR319C	'FET4	141.0	135.5	205.1	50.5	1.454	0.373	0.26
YOR032C	'HMS1	16.5	47.6	12.8	17.8	0.774	0.373	0.48
YLR438W	'CAR2	216.9	236.2	213.0	89.4	0.982	0.378	0.39
YBL078C	'ATG8	156.1	965.2	193.3	371.8	1.238	0.385	0.31
YKL163W	'PIR3	67.7	394.8	65.7	152.3	0.970	0.386	0.40
YMR271C	'URA10	63.8	385.4	52.7	153.1	0.826	0.397	0.48
YOR306C	'MCH5	125.6	370.7	116.8	148.7	0.930	0.401	0.43
YPR133W-A	'TOM5	3150.6	2352.9	4768.0	944.9	1.513	0.402	0.27
YDR074W	'TPS2	96.9	329.7	125.3	132.9	1.293	0.403	0.31
YLL026W	'HSP104	340.6	2125.6	345.9	872.0	1.016	0.410	0.40
YHR215W	'PHO12	26.3	289.5	94.1	118.8	3.579	0.410	0.11
YNL277W	'MET2	42.3	158.1	45.2	65.0	1.067	0.411	0.39
YFL021W	'GAT1	40.5	108.0	33.9	44.8	0.836	0.415	0.50
YEL059C-A	'SOM1	238.1	234.2	231.7	97.5	0.973	0.416	0.43
YDR481C	'PHO8	69.1	1500.8	135.9	641.6	1.968	0.427	0.22
YER037W	'PHM8	133.6	2285.7	163.6	985.5	1.224	0.431	0.35
YBR169C	'SSE2	76.7	1061.9	66.1	458.1	0.861	0.431	0.50
YML004C	'GLO1	254.4	887.4	267.4	391.7	1.051	0.441	0.42
YBR128C	'ATG14	20.7	62.8	21.8	27.8	1.052	0.443	0.42
YER091C	'MET6	101.2	160.8	159.2	71.3	1.573	0.443	0.28
YCR020C	'PET18	18.5	72.8	17.1	32.4	0.921	0.445	0.48
YPR093C	'ASR1	48.7	186.1	44.6	83.6	0.917	0.449	0.49
YLR303W	'MET17	86.0	302.7	194.2	136.0	2.258	0.449	0.20
YMR286W	'MRPL33	419.0	1005.5	498.4	460.1	1.190	0.458	0.38
YNL213C	'RRG9	104.7	324.2	120.6	148.8	1.152	0.459	0.40
YPR167C	'MET16	35.3	159.7	47.3	74.2	1.341	0.464	0.35
YPR184W	'GDB1	67.9	424.1	75.3	197.4	1.109	0.466	0.42
YDR171W	'HSP42	117.1	1426.1	127.1	665.6	1.086	0.467	0.43
YMR182C	'RGM1	113.6	329.5	110.9	154.7	0.976	0.470	0.48
YER002W	'NOP16	425.6	1013.0	457.6	476.7	1.075	0.471	0.44
YBR006W	'UGA2	81.3	243.3	89.0	115.1	1.094	0.473	0.43
YEL017C-A	'PMP2	2564.2	7044.1	5217.5	3348.9	2.035	0.475	0.23
YDR399W	'HPT1	879.6	330.8	1054.8	158.1	1.199	0.478	0.40
YHR211W	'FLO5	11.2	55.9	12.2	26.8	1.088	0.478	0.44
YDR502C	'SAM2	155.9	143.5	253.9	69.0	1.628	0.481	0.30
YHR104W	'GRE3	197.3	462.3	207.6	222.5	1.053	0.481	0.46
YLR251W	'SYM1	68.3	322.2	82.4	155.8	1.207	0.484	0.40
YDR482C	'CWC21	158.0	494.1	176.3	240.8	1.115	0.487	0.44
YML054C	'CYB2	207.9	113.6	263.6	55.4	1.268	0.487	0.38
YLR090W	'XDJ1	90.9	476.0	103.7	232.6	1.140	0.489	0.43
YBR280C	'SAF1	24.2	370.3	25.0	181.4	1.035	0.490	0.47
YNL274C	'GOR1	278.3	225.4	285.5	110.5	1.026	0.490	0.48
YJL141C	'YAK1	36.3	156.5	40.1	76.9	1.105	0.492	0.44
YCL058C	'FYV5	20.1	27.7	26.0	13.6	1.289	0.492	0.38
YPL111W	'CAR1	423.4	454.6	693.2	224.7	1.637	0.494	0.30
YER072W	'VTC1	970.1	4296.8	1203.3	2129.7	1.240	0.496	0.40
YML028W	'TSA1	2258.3	2911.9	2886.5	1449.3	1.278	0.498	0.39
YPL166W	'ATG29	28.9	116.0	34.1	58.3	1.177	0.502	0.43
YOR226C	'ISU2	84.2	624.4	146.5	313.6	1.740	0.502	0.29
YOL048C	'RRT8	77.6	334.8	88.5	169.6	1.140	0.506	0.44
YER042W	'MXR1	95.6	139.7	135.9	70.8	1.421	0.507	0.36
YPL123C	'RNY1	63.1	221.2	64.0	113.0	1.014	0.511	0.50

YHR136C	'SPL2	289.7	2523.5	383.8	1293.4	1.325	0.513	0.39
YOR317W	'FAA1	193.9	599.6	222.2	307.8	1.146	0.513	0.45
YBR047W	'FMP23	113.4	197.8	117.6	101.5	1.037	0.513	0.50
YHL036W	'MUP3	23.6	110.5	29.5	57.9	1.246	0.524	0.42
YKL035W	'UGP1	202.0	537.5	217.8	282.1	1.078	0.525	0.49
YMR261C	'TPS3	40.2	136.4	53.7	71.9	1.335	0.527	0.39
YHR106W	'TRR2	68.0	261.6	72.4	139.0	1.065	0.531	0.50
YDR058C	'TGL2	63.3	149.0	74.2	79.2	1.173	0.531	0.45
YOR120W	'GCY1	302.4	394.7	341.4	212.2	1.129	0.538	0.48
YJL142C	'IRC9	49.9	194.3	71.7	105.3	1.438	0.542	0.38
YCL018W	'LEU2	124.1	961.9	181.6	539.3	1.463	0.561	0.38
YLR189C	'ATG26	30.5	179.6	36.4	101.6	1.194	0.565	0.47
YLR068W	'FYV7	326.5	797.3	386.1	453.9	1.183	0.569	0.48
YMR136W	'GAT2	52.1	105.5	71.2	60.2	1.366	0.570	0.42
YDR033W	'MRH1	1305.5	3639.9	1865.2	2101.9	1.429	0.577	0.40
YDR379C-A	'SDH6	842.4	5146.1	1048.8	3006.6	1.245	0.584	0.47
YER054C	'GIP2	26.5	45.7	43.9	27.0	1.654	0.590	0.36
YHR171W	'ATG7	25.9	46.0	33.5	27.3	1.294	0.595	0.46
YDR228C	'PCF11	39.1	55.1	46.9	33.0	1.201	0.599	0.50
YGR215W	'RSM27	331.5	611.2	430.7	370.1	1.299	0.606	0.47
YEL049W	'PAU2	25.2	40.1	44.0	24.5	1.749	0.610	0.35
YML121W	'GTR1	153.1	299.5	189.3	186.4	1.236	0.622	0.50
YKL103C	'APE1	108.3	277.5	139.1	173.7	1.285	0.626	0.49
YLL009C	'COX17	845.7	2550.3	1476.0	1610.4	1.745	0.631	0.36
YDL045W-A	'MRP10	308.1	837.1	436.9	532.4	1.418	0.636	0.45
YPR151C	'SUE1	93.1	119.6	145.6	76.5	1.564	0.639	0.41
YPL240C	'HSP82	165.1	2965.7	304.6	1934.9	1.845	0.652	0.35
YDR258C	'HSP78	159.5	763.9	207.8	499.4	1.302	0.654	0.50
YJL164C	'TPK1	41.8	82.4	60.4	54.2	1.446	0.658	0.45
YNL065W	'AQR1	45.0	171.8	123.5	114.1	2.744	0.664	0.24
YCR024C-A	'PMP1	1942.5	3108.3	5804.8	2097.2	2.988	0.675	0.23
YJL057C	'IKS1	23.3	90.9	31.4	61.4	1.348	0.675	0.50
YIL053W	'GPP1	1054.4	4663.8	1681.1	3159.2	1.594	0.677	0.42
YHR174W	'ENO2	3182.9	20068.8	4834.3	13662.7	1.519	0.681	0.45
YDL160C-A	'MHF2	76.3	336.6	117.3	229.2	1.536	0.681	0.44
YGL009C	'LEU1	34.4	269.1	52.9	183.4	1.539	0.682	0.44
YJR009C	'TDH2	2234.9	12722.4	3299.3	8722.5	1.476	0.686	0.46
YOR193W	'PEX27	28.3	71.2	38.3	48.9	1.353	0.686	0.51
YMR180C	'CTL1	44.0	75.1	61.9	51.9	1.406	0.691	0.49
YOR027W	'STI1	253.6	1602.6	350.8	1116.1	1.383	0.696	0.50
YJR010W	'MET3	22.6	63.3	35.8	44.1	1.583	0.697	0.44
YGR142W	'BTN2	31.2	465.3	55.9	343.0	1.792	0.737	0.41
YLR109W	'AHP1	1424.7	5123.5	3060.1	3812.2	2.148	0.744	0.35
YCR018C	'SRD1	17.2	407.0	28.2	304.6	1.643	0.748	0.46
YNL042W	'BOP3	23.3	51.9	34.7	39.0	1.487	0.752	0.51
YKL086W	'SRX1	31.3	120.8	49.0	95.4	1.565	0.790	0.50
YLR301W	'HRI1	144.8	503.4	225.4	398.7	1.557	0.792	0.51
YOL136C	'PFK27	26.0	75.4	84.9	59.7	3.261	0.792	0.24
YPL213W	'LEA1	53.4	143.2	83.3	113.6	1.558	0.793	0.51
YNL191W	'DUG3	60.6	128.3	102.0	102.0	1.682	0.795	0.47
YDR263C	'DIN7	18.8	21.8	32.6	17.5	1.735	0.802	0.46
YJL153C	'INO1	14.7	12.8	26.5	10.4	1.801	0.815	0.45
YJL205C	'NCE101	618.5	626.3	1210.4	518.9	1.957	0.829	0.42
YPL189C-A	'COA2	224.7	245.9	397.0	208.7	1.767	0.849	0.48
YBR111W-A	'SUS1	118.4	560.7	229.5	512.1	1.939	0.913	0.47
YKR091W	'SRL3	36.2	132.7	71.2	124.6	1.970	0.939	0.48
YNL086W	'SNN1	60.9	281.2	116.7	269.1	1.916	0.957	0.50
YHL024W	'RIM4	26.0	14.6	58.3	14.0	2.241	0.959	0.43
YOR047C	'STD1	17.8	47.1	63.8	49.0	3.577	1.041	0.29
YJL217W	'REE1	2113.4	619.0	4953.2	646.1	2.344	1.044	0.45
YDR085C	'AFR1	15.9	34.8	33.7	37.0	2.121	1.064	0.50

YGL209W	*'MIG2*	31.8	116.1	227.7	133.2	7.152	1.147	0.16
YPR158W	*'CUR1*	18.7	166.8	55.3	198.6	2.960	1.190	0.40
YDR345C	*'HXT3*	35.9	80.1	175.8	199.8	4.901	2.494	0.51
YHR092C	*'HXT4*	18.9	63.2	348.9	341.3	18.449	5.405	0.29

Supplementary Table S3. Top 5 genes with upregulated expression with increased induction ratios in [*GAR*$^+$] cells.

Gene ID	Name	Description	[*gar*$^-$] /- GlcN (A)	[*gar*$^-$] /+ GlcN (B)	[*GAR*$^+$] /- GlcN (C)	[*GAR*$^+$] /+ GlcN (D)	Fold change (C/A)	Fold change (D/B)	Induction ratio (D/C)/(B/A)
YPL163C	*SVS1*	Cell wall and vacuolar protein; required for wild-type resistance to vanadate	223.8	10.8	182.2	114.8	0.814	10.628	13.06
YNL300W	*TOS6*	Glycosylphosphatidylinositol-dependent cell wall protein; expression is periodic and decreases in response to ergosterol perturbation or upon entry into stationary phase	263.8	21.6	258.4	177.1	0.979	8.187	8.36
YMR305C	*SCW10*	Cell wall protein with similarity to glucanases; may play a role in conjugation during mating based on mutant phenotype and its regulation by Ste12p	296.8	26.3	209.5	201.8	0.706	7.688	10.89
YNL301C	*RPL18B*	Ribosomal 60S subunit protein L18B; homologous to mammalian ribosomal protein L18	445.3	47.2	728.0	293.8	1.635	6.223	3.81
YMR011W	*HXT2*	High-affinity glucose transporter of the major facilitator superfamily; expression is induced by low levels of glucose and repressed by high levels of glucose	1143.7	79.6	3115.9	465.6	2.724	5.851	2.15

Supplementary Table S4. Top 5 genes with downregulated expression with increased induction ratios in [*GAR*$^+$] cells.

Gene ID	Name	Description	[*gar*$^-$] /- GlcN (A)	[*gar*$^-$] /+ GlcN (B)	[*GAR*$^+$] /- GlcN (C)	[*GAR*$^+$] /+ GlcN (D)	Fold change (C/A)	Fold change (D/B)	Induction ratio (D/C)/(B/A)
YAL040C	*CLN3*	G1 cyclin involved in cell cycle progression; activates Cdc28p kinase to promote G1 to S phase transition	127.5	32.0	45.8	28.5	0.359	0.891	2.48
YAL034W-A	*MTW1*	Essential component of the MIND kinetochore complex; critical to kinetochore assembly	34.4	12.8	14.8	13.0	0.431	1.021	2.37
YBL002W	*HTB2*	Histone H2B; core histone protein required for chromatin assembly and chromosome function	2939.7	1182.9	1589.0	1323.8	0.541	1.119	2.07
YBL003C	*HTA2*	Histone H2A; core histone protein required for chromatin assembly and chromosome function	3344.0	2052.4	1868.8	2348.8	0.559	1.144	2.05
YBR050C	*REG2*	Regulatory subunit of the Glc7p type-1 protein phosphatase; involved with Reg1p, Glc7p, and Snf1p in regulation of glucose-repressible genes	88.1	39.9	49.8	47.3	0.565	1.185	2.10

Supplementary Table S5. Top 5 genes with upregulated expression with decreased induction ratios in [*GAR*$^+$] cells.

Gene ID	Name	Description	[*gar*$^-$] /- GlcN (A)	[*gar*$^-$] /+ GlcN (B)	[*GAR*$^+$] /- GlcN (C)	[*GAR*$^+$] /+ GlcN (D)	Fold change (C/A)	Fold change (D/B)	Induction ratio (D/C)/(B/A)
YHR092C	*HXT4*	High-affinity glucose transporter; expression is induced by low levels of glucose and repressed by high levels of glucose	18.9	63.2	348.9	341.3	18.449	5.405	0.29
YDR345C	*HXT3*	Low affinity glucose transporter of the major facilitator superfamily; expression is induced in low or high glucose conditions	35.9	80.1	175.8	199.8	4.901	2.494	0.50
YPR158W	*CUR1*	Sorting factor, central regulator of spatial protein quality control; involved in destabilization of [*URE3*] prions	18.7	166.8	55.3	198.6	2.960	1.190	0.40
YGL209W	*MIG2*	Zinc finger transcriptional repressor; cooperates with Mig1p in glucose-induced gene repression	31.8	116.1	227.7	133.2	7.152	1.147	0.16
YDR085C	*AFR1*	Protein required for pheromone-induced projection (shmoo) formation; regulates septin architecture during mating	15.9	34.8	33.7	37.0	2.121	1.064	0.50

Supplementary Table S6. Top 5 genes with downregulated expression with decreased induction ratios in [*GAR*$^+$] cells.

Gene ID	Name	Description	[*gar*$^-$] /- GlcN (A)	[*gar*$^-$] /+ GlcN (B)	[*GAR*$^+$] /- GlcN (C)	[*GAR*$^+$] /+ GlcN (D)	Fold change (C/A)	Fold change (D/B)	Induction ratio (D/C)/(B/A)
YPL223C	*GRE1*	Hydrophilin essential in desiccation-rehydration process; regulated by the HOG pathway	297.1	3521.2	36.6	183.2	0.123	0.052	0.42
YMR175 W	*SIP18*	Phospholipid-binding hydrophilin; expression is induced by osmotic stress	10528.0	17046.6	2211.5	992.3	0.210	0.058	0.28
YGR256W	*GND2*	6-phosphogluconate dehydrogenase; catalyzes an NADPH regenerating reaction in the pentose phosphate pathway	107.6	476.8	40.8	31.7	0.380	0.066	0.17
YMR169C	*ALD3*	Cytoplasmic aldehyde dehydrogenase; expression is induced by stress and repressed by glucose	94.4	659.5	44.1	52.8	0.467	0.080	0.17
YMR174C	*PAI3*	Cytoplasmic proteinase A (Pep4p) inhibitor; dependent on Pbs2p and Hog1p protein kinases for osmotic induction	6149.8	23619.7	1717.0	1939.6	0.279	0.082	0.29
